# Supplementary material for: Polyketide synthase gene clusters in the ericoid mycorrhizal fungus Oidiodendron maius: genome-wide prediction, expression profiling, and functional characterization of knockout mutants
Source: Front Plant Sci. 2026 Sep 16;17:1924430. doi: 10.3389/fpls.2026.1924430 (PMC13623903; doi:10.3389/fpls.2026.1924430)
Supplement: Supplementary file 1 [file SupplementaryFile1.zip › Supplementary Materials/Supplementary Folder 1.zip!/Oidma1_AssemblyScaffolds_Repeatmasked/knownclusterblast/cluster11/jgi.p_Oidma1_157855_mibig_hits.html]

| MIBiG Protein | Description | MIBiG Cluster | MiBiG Product | % ID | % Coverage | BLAST Score | E-value |
| --- | --- | --- | --- | --- | --- | --- | --- |
| EAL85111.2 | cytochrome\_P450\_oxidoreductase,\_putative | BGC0001037 | Pseurotin\_A\_biosynthetic\_gene\_cluster | 30 | 87.3267326733 | 173 | 4.9e-43 |
| AAS90061.1 | OrdA | BGC0000009 | Aflatoxin\_biosynthetic\_gene\_cluster | 25 | 97.8217821782 | 144 | 4.1e-34 |
| AAS90013.1 | OrdA | BGC0000007 | Aflatoxin\_biosynthetic\_gene\_cluster | 29 | 62.1782178218 | 123 | 7.6e-28 |
| BAH23999.1 | cytochrome\_P450 | BGC0000356 | Fumitremorgin\_biosynthetic\_gene\_cluster | 36 | 30.8910891089 | 94 | 2.9e-19 |
| CYP82X1 |  | BGC0001325 | Noscapine\_biosynthetic\_gene\_cluster. | 27 | 42.3762376238 | 67 | 4.9e-11 |
| BAI44340.1 | P450 | BGC0000677 | Cyclooctatin\_biosynthetic\_gene\_cluster | 28 | 33.4653465347 | 66 | 1.1e-10 |
| AAK33073.1 | cytochrome\_P450 | BGC0001278 | Nivalenol\_/\_deoxynivalenol\_/\_3-acetyldeoxynivalenol\_/ | 32 | 26.3366336634 | 61 | 2.7e-09 |
| Manes.12G132900 |  | BGC0001318 | Linamarin\_/\_Lotaustralin\_biosynthetic\_gene\_cluster. | 26 | 25.9405940594 | 60 | 7.9e-09 |
| ACH72911.1 | AflV | BGC0000011 | Aflatoxin/sterigmatocystin\_biosynthetic\_gene\_cluster | 22 | 86.7326732673 | 57 | 6.6e-08 |
| CYP719A21 |  | BGC0001325 | Noscapine\_biosynthetic\_gene\_cluster. | 26 | 23.9603960396 | 56 | 1.1e-07 |
| chr3.CM0292.110.r2.m |  | BGC0001317 | Lupeol\_biosynthetic\_gene\_cluster. | 28 | 29.702970297 | 56 | 1.1e-07 |
